# Apolipoprotein C1 -317H1/H2 and the rs4420638 genetic variations and risk of gestational diabetes mellitus in Chinese women: a case-control study

**DOI:** 10.3389/fendo.2025.1681268

**Published:** 2025-11-21

**Authors:** Wandi Ma, Linbo Guan, Xinghui Liu, Yujie Wu, Zhengting Zhu, Yuwen Guo, Ping Fan, Huai Bai

**Affiliations:** 1 Laboratory of Genetic Disease and Perinatal Medicine, West China Second University Hospital, Sichuan University, Chengdu, Sichuan, China; 2 Key Laboratory of Birth Defects and Related Diseases of Women and Children of the Ministry of Education, West China Second University Hospital, Sichuan University, Chengdu, Sichuan, China; 3 Department of Obstetrics and Gynecology, West China Second University Hospital, Sichuan University, Chengdu, Sichuan, China

**Keywords:** APOC1 gene, single nucleotide polymorphism, gestational diabetes mellitus, atherometabolic traits, dyslipidemia

## Abstract

**Background:**

Dyslipidemia and oxidative stress are key components in the pathophysiology of gestational diabetes mellitus (GDM), yet the contribution of genetic factors to these metabolic disturbances remains unclear. This study aimed to investigate the relationship between two lipid-related genetic polymorphisms, apolipoprotein C1 (apoC1) gene -317H1/H2 (rs1568822) and rs4420638, with GDM risk and lipid profiles and oxidative stress markers in Chinese populations.

**Methods:**

The apoC1 -317H1/H2 and rs4420638 polymorphisms were genotyped in 734 GDM patients and 1,102 control subjects. Genetic association with GDM risk and related traits were also analyzed,

**Results:**

The distribution of genotype and allele in both polymorphisms were similar between the two groups. However, the combined H1H1/AG+GG genotype was significantly more frequent in women with GDM than in the control group. GDM patients who carried H1H1/AG+GG genotype were 1.97-fold increased risk to develop GDM (95% CI: 1.140-3.414, *P* = 0.015). H2 allele correlated with decreased levels of low-density lipoprotein cholesterol (LDL-C), apoB and lower atherogenic index (AI) in both groups, in addition to the GDM group also with lower total cholesterol (TC), whereas the G allele of rs4420638 correlated with increased triglyceride and decreased apoA1 levels.

**Conclusion:**

ApoC1 gene polymorphisms associate with GDM risk and affect the lipid profile. The combined H1H1/AG+GG genotype of the apoC1 gene polymorphisms appears to augment the propensity to develop GDM, while the rs4420638 polymorphism links to adverse lipid components in the patients. Further genetic studies to add information beyond the traditional risk factors in GDM and to identify risk genotypes will help in early prediction and identification of at-risk patients.

## 1 Introduction

Gestational diabetes mellitus (GDM) is a common pregnancy complication characterized by the onset of hyperglycemia during gestation (1). Its prevalence varies worldwide, affecting 1–28% of pregnancies, depending on diagnostic criteria and population characteristics (2). Rising obesity rates, advanced maternal age, sedentary lifestyles, and suboptimal nutrition contribute to the increasing global incidence of GDM (3, 4). It poses immediate risks to both mother and fetus during pregnancy and delivery and is associated with long-term health issues such as elevated risk of type 2 diabetes mellitus (T2DM) and cardiovascular disease (4, 5). GDM shares a common pathology with T2DM, with pregnancy serving as a stressor that unmask hyperglycemia during pregnancy among those who are at risk of future diabetes because of impaired β-cell function (6).

GDM belongs to a disease with a complex pathogenesis, mainly attributing to genetic and environmental risk factors. Among the risk factors, genetic factor is the important one for GDM. To date, genetic susceptibility to GDM has been widely studied using candidate genes and GWAS approaches. While the association of genes with risk of GDM (7–9) and related SNPs have been widely explored as molecular biomarkers for GDM (10), and novel SNPs have continued to emerge, especially with the wave of GWAS approach, in the recent decade (6, 11, 12), their role needs definitely established. Although the exact mechanism underlying GDM are not fully understood, factor like lipid imbalance in addition to other factors are believed to play contributing roles. Indeed, several studies reveal that the changes in maternal lipid metabolism are closely related the role of hormones and onset of insulin resistance during gestation (13, 14), and increased levels of estrogen, progestrogen, and insulin facilitate lipid production and storage during early pregnancy (13–15). Several studies have demonstrated that dyslipidemia significantly influences the development of GDM (16–19). Therefore, the genes linked to regulate lipid metabolism might be the candidate genes that potentially related to the genetic predisposing effects on GDM development. Up to now, few studies have established an association with GDM, and no study has investigated a relationship between *apolipoprotein C1 (apoC1)* genetic variants and GDM development.

The *apoC1* gene is located on chromosome 19q13.32. A well-studied polymorphism, -317H1/H2 (rs11568822), involves a 4-base pair (bp) CGTT deletion/insertion (Del/Ins) located 317 bp upstream of the *apoC1* transcription starting site on chromosome 19. *In vitro*, the insertion (Ins) allele promotes higher *apoC1* transcription than the Del allele (20). According to Shachter et al. (21), the Ins allele of rs11568822 is correlated with reduced circulating apoC1 levels, but this relationship was not confirmed by Cohn et al. (22). In general, *in vitro* and animal studies suggest that apoC1 is an important regulator of lipid metabolism. However, evidence from human studies remains limited and mainly comes from association analyses based on very small cohorts. Such studies have reported that individuals with the Del allele tend to exhibit elevated serum triglyceride (TG) levels compared with heterozygous or non-carriers (20, 21, 23).

Another SNP, rs4420638, an A→G transition of nucleotide, located about 340 bp downstream of the 3’ terminus of the *apoC1* gene, tags a linkage desequibrium (LD) block involving the *TOMM40*, *apo E*, and *apoC1* genes, and has been implicated in dyslipidemia (24). The rs4420638 polymorphism has been associated with the activity of lipoprotein-associated phospholipase A2 (Lp-PLA2), and TC, TG, LDL-C and HDL-C (25–29). It has been linked to a higher risk of metabolic syndrome, T2DM, and coronary heart disease (28).

Given that *apoC1* is implicated in the lipid metabolism (30–32), inflammation (33), and possibly oxidative stress function (34), all of these are known critical components for the pathogenesis involved in GDM, and several studies have identified the two polymorphisms as dyslipidemia- and/or cardiometabolic diseases-linked variants, it is possible that the *apoC1* polymorphisms with functional features may influence GDM development and/or associated metabolic traits or phenotypes. However, the potential link between *apoC1* gene polymorphisms and GDM remains unclear.

Considering the results for *apoC1* -317H1/H2 and rs4420688 as lipid and/or cardiometabolic disease-associated variants, and the total lack of studies exploring the association of these genetic variants with the risk of GDM, we conducted the present study by investigating whether *apoC1–*317 H1/H2 and rs4420638 polymorphisms were associated with GDM. We examined a well-defined Chinese cohort using a case-control design with a substantial sample size (734 patients with GDM and 1,102 controls). We also evaluated whether these polymorphisms were linked to participant traits.

## 2 Materials and methods

### 2.1 Study participants

This study included 734 patients with GDM and 1,102 controls aged 17–44 years from the Obstetrics Department of the West China Second University Hospital of Sichuan University, Chengdu, China. All participants provided informed consent and the Institutional Review Board approved the study protocol (Approval No. 2017-033). The study was conducted according to the principles of the Declaration of Helsinki.

GDM was diagnosed according to the criteria established by the International Association of the Diabetes and Pregnancy Study Groups (IADPSG) (35). Control women with uncomplicated pregnancies were recruited from hospital departments during the same period. During selection, we frequently matched controls to cases by strata according to maternal and gestational age, ethnicity (Han Chinese), parity, date of delivery, and distance from the hospital.

The participants were free from overt chronic or acute illnesses, including infections, neoplasms, thyroid disorders, and cardiovascular diseases.

We then calculated the sample size for this study. A minimum of 300 patients and 300 controls were required, based on the results of a report on the *apoC1* -317H1/H2 (HpaI, rs1568822) polymorphism in a case-control study on alzheimer disease in Taiwan (36) (the A [H2] allele frequencies for cases and controls were 0.298 and 0.168, respectively, power = 0.80, significance level= 0.05) according to the Power and Sample Size Calculations (PS) Program version 3.1.6. In this study, for the genetic association study of the *apoC1* SNPs, 734 patients and 1,102 controls were included.

The demographic data collected included age, height, weight, resting systolic blood pressure (SBP), and diastolic blood pressure (DBP) measured between 24 and 28 weeks of gestation during the first pregnancy, along with newborn length and weight at birth. Pre-pregnancy BMI and that at delivery were calculated as weight (kg) divided by height squared (m²).

Blood samples were collected in the third trimester or before delivery after at least 8 h of fasting. Plasma and serum were separated at 4°C within 2 h and preserved at − 80°C until analysis.

### 2.2 DNA extraction and genotyping

Genomic DNA was extracted from 500 μL sample of peripheral blood according to the Erlich method. Genotyping of *apoC1* polymorphisms rs4420638 and -317H1/H2 was performed using a TaqMan allelic discrimination assay based on real-time PCR and PCR-restriction fragment length polymorphism methods, respectively.

The rs4420638 polymorphism was genotyped using a TaqMan allelic discrimination assay to assess a 101-bp PCR-amplified fragment. Primers were: forward 5’-TCAGCCTAGCAATGTCACTATGC-3’ and reverse 5’-GTCTGCCTCAAAACA AAACAAAA-3’. The probes used were wild-type HEX-CTTTTCCTaGTGTGGTCT A-TAMRA and mutant FAM-CACTTTTCCTgGTGTGGT-TAMRA. Synthetic 151-bp DNA fragments representing the wild-type (A) and mutant (G) alleles of *apoC1* were used as positive controls. PCR amplification was conducted on an iCycler iQ real-time PCR detection system (Bio-Rad Laboratories, Hercules, CA, USA) under the following conditions: initial denaturation at 95°C for 3 min, followed by 42 cycles of denaturation at 95°C for 15 s, annealing at 55°C for 20 s, and extension at 72°C for 15 s.

Fluorescence was recorded at the end of each cycle and the final endpoint. Primers, probes, and controls were designed and synthesized by TSINGKE Biotech Co., Ltd. (Chengdu, China).

For the -317H1/H2 genotyping, the 221-bp (H1, CGTT deletion) and 225-bp (H2, CGTT insertion) fragments were amplified using the forward primer 5′-TTTGAGCTCGGCTCTTGAACAGGAA-3′ and the reverse primer 5′-GGTCCCGGGCACTTCCCTTAGCCCCA-3′ (37). PCR products of the -317H1/H2 polymorphism were digested with HpaI (Thermo Fisher Scientific, Waltham, MA, USA), electrophoretically separated on a 3.0% agarose gel, and visualized using Genecolor fluorescent staining. Enzymatic digestion produced 159- and 66-bp fragments for the -317H2 allele and a non-digested 221-bp fragment for the -317H1 allele.

For the purpose of quality control of the genotyping, 30% of duplicate DNA samples were included. Genotyping call rates for -317H1/H2 and rs4420638 polymorphism were 99.4% and 99.1%, respectively, and error rates were 1.6% and 5.2%, respectively.

### 2.3 Analysis of biochemical parameters

Biochemical parameters were measured using standard laboratory techniques. Plasma insulin levels were quantified using a chemiluminescence assay (IMMULITE 2000; Diagnostic Products Corp., Los Angeles, CA, USA). Total cholesterol (TC), high-density lipoprotein cholesterol (HDL-C), and triglyceride (TG) levels were determined enzymatically (Boehringer, Mannheim, Germany). Serum apoA1 and apoB levels were measured using a polyethylene glycol-enhanced immunoturbidimetric assay (Siemens Healthcare Diagnostics, Munich, Germany) on a Hitachi 7600–010 automated analyzer (Hitachi, Tokyo, Japan). Plasma glucose levels were assessed using the glucose oxidase method (Roche Diagnostics, Basel, Switzerland). The concentrations of serum malondialdehyde and total antioxidant capacity were measured using respective kits from the Nanjing Jiancheng Bioengineering Institute (Nanjing, China). Serum total oxidant status was determined using a microplate colorimetric method established in our laboratory (38). The oxidative stress index (arbitrary units) was expressed as the ratio of total oxidant status to total antioxidant capacity. The atherogenic index (AI) was calculated as (TC – HDL-C)/HDL-C. Insulin resistance was evaluated using the homeostatic model assessment for insulin resistance test, calculated as fasting glucose (mmol/L) × fasting insulin (μU/mL) divided by 22.5. For all determinations, intra-assay variations were < 5% and inter-assay variations were < 10%.

### 2.4 Statistical analysis

Data are presented as mean ± standard deviation. The allele frequencies of *apoC1* polymorphisms were determined by direct counting. The Hardy-Weinberg equilibrium was assessed using the chi-square test in both groups. Genotype and allele frequencies were compared between the GDM and control groups using chi-square analysis. Differences in continuous variables between the groups were analyzed using independent-sample Student’s t-tests. An analysis of covariance (ANCOVA) or a two way analysis of variance (ANOVA) was used to estimate the differences in clinical parameters, hormonal levels, and oxidative stress markers between the two groups or genotype subgroups after correction for differences in maternal age, pre-pregnancy BMI, gestational age at sampling, and fasting insulin and glucose levels. Statistical significance was set at *P* < 0.05. Statistical analyses were conducted using SPSS 21.0 (IBM Corp., Armonk, NY, USA).

## 3 Results

### 3.1 Participant clinical and biochemical profiles

As shown in **Table 1**, the pre-pregnancy BMI and gestational age at sampling were higher in the GDM group than in the control group. Of the 734 women with GDM, 84 were treated with insulin while the remainder were managed with dietary and exercise modifications.

**Table 1:** Clinical characteristics and metabolic and oxidative stress parameters in GDM patients and control women.

	GDM (n=734)	Controls (n=1102)	*P*	*P^a^*
Clinical characteristics				
Age (years)	35.56 ± 4.04	35.49 ± 3.72	0.710	
Pre-pregnancy BMI (kg/m^2^)	22.24 ± 2.93	21.22 ± 2.69	0.000	
Gestational age at sampling(week)	36.92 ± 3.23	36.04 ± 3.53	0.000	
Gestational age at delivery(week)	38.89 ± 1.80	39.24 ± 0.88	0.000	0.000
Delivery BMI(Kg/m^2^)	26.80 ± 3.17	26.71 ± 2.71	0.546	0.000
Gestational weight gain(Kg)	11.49 ± 4.21	14.01 ± 4.30	0.000	0.000
SBP (mmHg)	115.71 ± 11.80	115.07 ± 10.09	0.228	0.156
DBP (mmHg)	72.69 ± 9.03	72.11 ± 7.97	0.179	0.132
OGTT-0h (mmol/L) ^b^	4.89 ± 0.54	4.43 ± 0.29	0.000	0.000
OGTT-1h (mmol/L) ^b^	9.90 ± 1.40	7.46 ± 1.29	0.000	0.000
OGTT-2h (mmol/L) ^b^	8.70 ± 1.34	6.54 ± 1.02	0.000	0.000
Newborn birth length(cm)	49.61 ± 1.84	49.86 ± 1.90	0.005	0.001
Newborn birth weight(g)	3335.80 ± 444.68	3379.34 ± 374.34	0.030	0.000
Macrosomia % (n)	5.45 (40)	4.17 (46)	0.205	
Insulin treatment (n)	11.44 (84)	0 (0)		
Metabolic profile ^△^				
Fasting Ins (pmol/L)	104.20 ± 126.00	72.37 ± 35.45	0.000	0.000 ^c^
Fasting Glu (mmol/L)	4.62 ± 0.73	4.35 ± 0.43	0.000	0.000 ^c^
HOMA-IR	3.39 ± 5.56	2.04 ± 1.08	0.000	0.045
TG (mmol/L)	3.90 ± 1.68	3.63 ± 1.41	0.001	0.036
TC (mmol/L)	5.96 ± 1.10	6.07 ± 1.09	0.039	0.201
HDL-C (mmol/L)	1.97 ± 0.44	1.99 ± 0.41	0.273	0.397
LDL-C (mmol/L)	2.98 ± 0.97	3.18 ± 0.99	0.000	0.002
TG/HDL	2.10 ± 1.12	1.91 ± 0.88	0.000	0.011
AI	2.10 ± 0.57	2.12 ± 0.60	0.486	0.778
ApoA1 (g/L)	2.30 ± 0.42	2.38 ± 0.43	0.000	0.001
ApoB (g/L)	1.15 ± 0.26	1.15 ± 0.26	0.889	0.609
Oxidative stress parameters ^△△^				
TOS (μmol H2O2 Equiv./L)	25.83 ± 10.56	21.11 ± 7.02	0.000	0.000
TAC (mmol Trolox Equiv./L)	1.12 ± 0.21	1.11 ± 0.19	0.277	0.079
TOS/TAC	23.32 ± 10.11	19.53 ± 7.33	0.000	0.000
MDA (μmol/L)	5.88 ± 1.42	5.37 ± 1.21	0.000	0.000

Values are presented as mean ± standard deviation.

BMI: body mass index; SBP: systolic blood pressure; DBP: diastolic blood pressure; OGTT: oral glucose tolerance test; Ins: insulin; Glu: glucose; HOMA-IR: homeostatic model assessment of insulin resistance; TG: triglycerides; TC: total cholesterol; HDL-C: high-density lipoprotein cholesterol; LDL-C: low-density lipoprotein cholesterol; AI: atherogenic index; ApoA1: apolipoprotein A1; ApoB: apolipoprotein B; TOS: total oxidant status; TAC: total antioxidant capacity; MDA: malondialdehyde.

*P^a^* All comparisons of parameters were corrected for differences in age, pre-pregnancy BMI and gestational age at sampling, fasting Ins, and fasting Glu between the two groups, except for the parameters of age, pre-pregnancy BMI, and gestational age at sampling; ^b^: plasma glucose levels during OGTT between 24 and 28 weeks of gestation; ^c^: comparisons of fasting Ins and fasting Glu were corrected for differences in age, pre-pregnancy BMI, and gestational age at sampling.

^△^: GDM: 693; Control: 1040; ^△△^: GDM: 571; Control: 815.

Differences in maternal pre-pregnancy BMI, gestational age at the time of sampling, and insulin treatment between GDM and control participants may confound the comparisons of metabolic profiles and oxidative stress parameters. Therefore, all relevant intergroup comparisons were adjusted to account for maternal age, pre-pregnancy BMI, gestational age at sampling, and fasting insulin and glucose levels.

Compared with the control group, the GDM group exhibited significantly lower gestational age at delivery, gestational weight gain, neonatal birth length and weight, as well as LDL-C and apoA1 levels. In contrast, fasting-, 1-h, and 2-h glucose levels during the oral glucose tolerance test (performed between 24 and 28 weeks of gestation), along with TG/HDL-C, apoB/apoA1, fasting insulin and glucose, homeostatic model assessment for insulin resistance, malondialdehyde, total oxidant status, and oxidative stress index (TOS/TAC), were markedly increased in the GDM group (*P* < 0.05–0.001).

### 3.2 Distribution of the apoC1 genotype and allele frequencies

Genotyping results for the rs4420638 and the -317H1/H2 polymorphisms of *apoC1* show in **Supplementary Figures S1**, **S2**, respectively. In both the GDM and control participants, the genotypes of the two polymorphisms were in Hardy-Weinberg equilibrium. The frequencies of the *apoC1* genotypes and alleles in these polymorphisms showed no notable differences between the GDM and control groups (*P* > 0.05, **Table 2**).

**Table 2:** Frequencies of *apoC1 rs4420638* and *apoC1 -317H1/H2* genotypes and alleles in GDM patients compared to control women.

		GDM (n=734)	Controls (n=1102)	*X^2^*	P
Genotype					
apoC1 -317H1/H2	H1H1	482 (65.7%)	694 (63.0%)		
	H1H2	222 (30.2%)	364 (33.0%)		
	H2H2	30 (4.1%)	44 (4.0%)	1.579	0.454
apoC1 rs4420638	AA	556 (75.7%)	820 (74.4%)		
	AG	168 (22.9%)	262 (23.8%)		
	GG	10 (1.4%)	20 (1.8%)	0.805	0.669
Allele					
apoC1 -317H1/H2	H1	593 (80.8%)	876 (79.8)		
	H2	141 (19.2%)	222 (20.2)	0.282	0.596
apoC1 rs4420638	A	640 (87.2%)	951 (86.3%)		
	G	94 (12.8%)	151 (13.7%)	0.306	0.580

Data of genotype are presented as number (%) of patients or controls.

### 3.3 Analysis of combined genotypes of the apoC1 polymorphisms

We evaluated the correlation between the combined genotypes of *apoC1* -317H1/H2 and rs4420638, and GDM. Due to the small sample size of homozygous H2H2 at -317H1/H2 and GG at rs4420638, we combined them with the respective heterozygotes of the two polymorphisms for combination analysis. The combined genotype frequency of H1H1/AG+GG in patients with GDM (4.8%) was significantly higher than that in the control group (3.7%; odds ratio (OR) = 1.973, 95% confidence interval (CI) = 1.140–3.414, *P* = 0.015) after adjusting for age, pre-pregnancy BMI, gestational age at sampling, fasting insulin and glucose levels, and insulin use (**Table 3**). The frequencies of the other genotype combinations did not differ between the GDM and control groups (*P* > 0.05, **Table 3**).

**Table 3:** Frequencies of combined genotypes of *apoC1 rs4420638* and *apoC1 -317H1/H2* in patients in the GDM and control groups.

Genotype combinations	GDM (n=734)	Controls (n=1102)	OR	95%CI	*P*
*H1H1/AA*	446 (60.8%)	653 (59.3%)	1.000	–	–
*H1H1/AG+GG*	35 (4.8%)	41 (3.7%)	1.973	1.140-3.414	0.015
*H1H2+H2H2/AA*	110 (15.0%)	167 (15.2%)	1.095	0.799-1.501	0.572
*H1H2+H2H2/AG+GG*	143 (19.5%)	241 (21.9%)	0.957	0.719-1.275	0.766

Data on genotype combinations are presented as number (%) of patients or controls.

Odds ratios (ORs) and 95% confidence intervals (CIs) were corrected for differences in age, pre-pregnancy BMI, gestational age at sampling, fasting Ins, fasting Glu, insulin use (+, -), and calculated using a multinomial logistic regression model, with the *H1H1/AA* combined genotypes (wild-type) as the reference category.

### 3.4 Effects of polymorphic sites of apoC1 gene on clinical and biochemical variables

**Figure 1:**
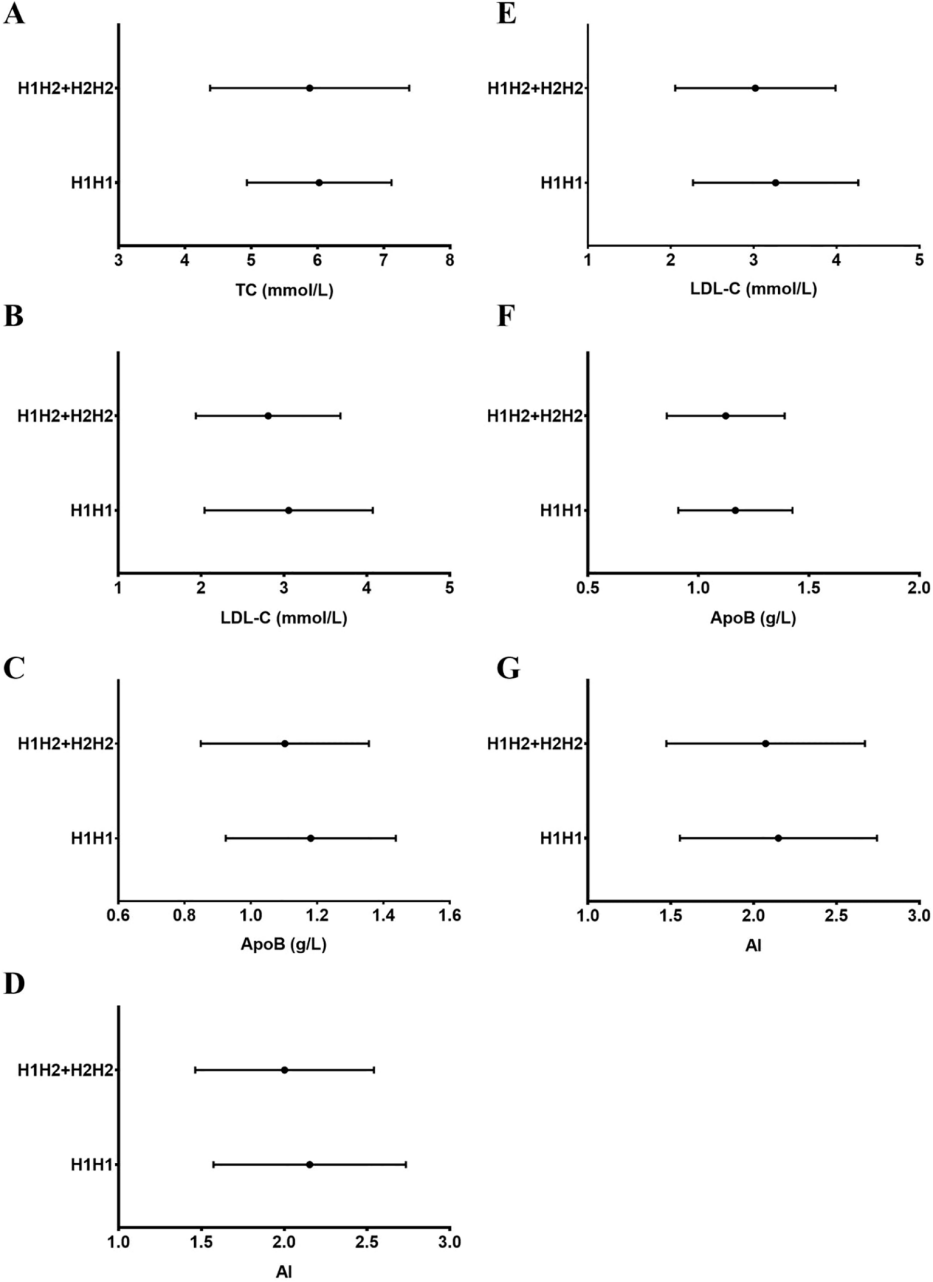
Total cholesterol (TC), low-density lipoprotein cholesterol (LDL-C), and ApoB levels, as well as the atherogenic index (AI), according to the -317 H1H2 apoC1 polymorphism (allele carrier status). A significant decrease in TC **(A)**, LDL-C **(B)**, ApoB **(C)**, and AI **(D)** in the GDM group (P = 0.042, 0.001, 0.001 and 0.001, respectively), and in LDL-C **(E)**, ApoB **(F)**, and AI **(G)** in the control group (P = 0.006, 0.032, and 0.019, respectively), was identified in H2-allele (H1H2 + H2H2) carriers compared with H1H1 genotype carriers. The graphs show mean values ± standard deviation (p < 0.05–0.001, by multivariable analysis).

After correcting for age, pre-pregnancy BMI, gestational age at sampling, and fasting insulin and glucose levels, GDM subjects with the H2 allele (H1H2 + H2H2) at the -317H1/H2 polymorphism showed decreased TC, LDL-C, apoB, and AI compared to those with the H1H1 genotype (*P* < 0.05–0.001, **Table 4**, **Figures 1A-D**). The influence of the H2 allele on LDL-C, ApoB, and AI levels was also pronounced in the control group with normal pregnancies (*P* < 0.05–0.01, **Table 4**, **Figures 1E-G**).

**Table 4:** Clinical characteristics, metabolic and oxidative stress parameters of the *apoC1 -317H1/H2* genotypes in GDM patients and control women.

	GDM		Controls	
	*H1*H1 (n=482)	*H1H2+H2H2* (n=222 + 30)	*H1H1* (n=694)	*H1H2+H2H2* (n=364 + 44)
Age (years)	35.41 ± 4.08	35.85 ± 3.95	35.46 ± 3.67	35.53 ± 3.82
Pre-pregnancy BMI (kg/m^2^)	22.30 ± 2.97	22.11 ± 2.86	21.28 ± 2.61	21.11 ± 2.83
Gestational age at sampling (week)	37.03 ± 3.19	36.72 ± 3.29	36.01 ± 3.54	36.09 ± 3.53
Delivery BMI(kg/m^2^)	26.92 ± 3.29	26.58 ± 2.93	26.75 ± 2.78	26.65 ± 2.60
Metabolic profile ^△^				
Fasting Ins (pmol/L)	106.70 ± 132.54	99.18 ± 112.28	70.76 ± 35.45	75.36 ± 35.45
Fasting Glu (mmol/L)	4.61 ± 0.73	4.65 ± 0.72	4.34 ± 0.44	4.37 ± 0.41
HOMA-IR	3.47 ± 5.83	3.22 ± 5.00	1.99 ± 1.08	2.12 ± 1.09
TG (mmol/L)	3.94 ± 1.77	3.81 ± 1.51	3.59 ± 1.46	3.70 ± 1.33
TC (mmol/L)	6.02 ± 1.09	5.84 ± 1.12 *	6.11 ± 1.08	6.02 ± 1.09
HDL-C (mmol/L)	1.96 ± 0.43	1.99 ± 0.46	1.98 ± 0.40	2.01 ± 0.43
LDL-C (mmol/L)	3.06 ± 1.02	2.82 ± 0.87 **	3.27 ± 1.00	3.03 ± 0.97 **
TG/HDL	2.14 ± 1.20	2.02 ± 0.95	1.89 ± 0.90	1.94 ± 0.84
AI	2.15 ± 0.58	2.00 ± 0.52 **	2.15 ± 0.59	2.07 ± 0.60 *
ApoA1 (g/L)	2.30 ± 0.43	2.30 ± 0.39	2.38 ± 0.45	2.38 ± 0.41
ApoB (g/L)	1.18 ± 0.26	1.10 ± 0.25 **	1.17 ± 0.26	1.12 ± 0.27 *
Oxidative stress parameters^△△^				
TOS (μmol H_2_O_2_ Equiv./L)	25.89 ± 10.25	25.73 ± 11.15	21.08 ± 7.12	21.14 ± 6.86
TAC (mmol Trolox Equiv./L)	1.13 ± 0.21	1.10 ± 0.20	1.10 ± 0.19	1.11 ± 0.20
TOS/TAC	23.06 ± 10.00	23.79 ± 10.33	19.59 ± 7.43	19.42 ± 7.17
MDA (μmol/L)	5.87 ± 1.38	5.89 ± 1.49	5.32 ± 1.21	5.46 ± 1.22

Values are presented as mean ± standard deviation.

BMI: body mass index; SBP: systolic blood pressure; DBP: diastolic blood pressure; OGTT: oral glucose tolerance test; Ins: insulin; Glu: glucose; HOMA-IR: homeostatic model assessment of insulin resistance; TG: triglycerides; TC: total cholesterol; HDL-C: high-density lipoprotein cholesterol; LDL-C: low-density lipoprotein cholesterol; AI: atherogenic index; ApoA1: apolipoprotein A1; ApoB: apolipoprotein B; TOS: total oxidant status; TAC: total antioxidant capacity; MDA: malondialdehyde. All parameter comparisons were corrected for differences in age, pre-pregnancy BMI, gestational age at sampling, and fasting Ins and Glu levels between the two groups, except for age, pre-pregnancy BMI, and gestational age at sampling (GDM group was also corrected in insulin use).

^△^: GDM: *H1H1*: 452, *H1H2+H2H2*: 211 + 30; Control: *H1H1*: 660, *H1H2+H2H2*: 343 + 37; ^△△^: GDM: *H1H1*: 374, *H1H2+H2H2*: 174 + 23; Control: *H1H1*: 512, *H1H2+H2H2*: 271 + 32.

*: The *AG+GG* genotypes, compared with the *AA* genotype in the same group (GDM group or control group), P<0.05; **: The *AG+GG* genotypes, compared with the *AA* genotype in the same group (GDM group or control group), P<0.01.

**Figure 2:**
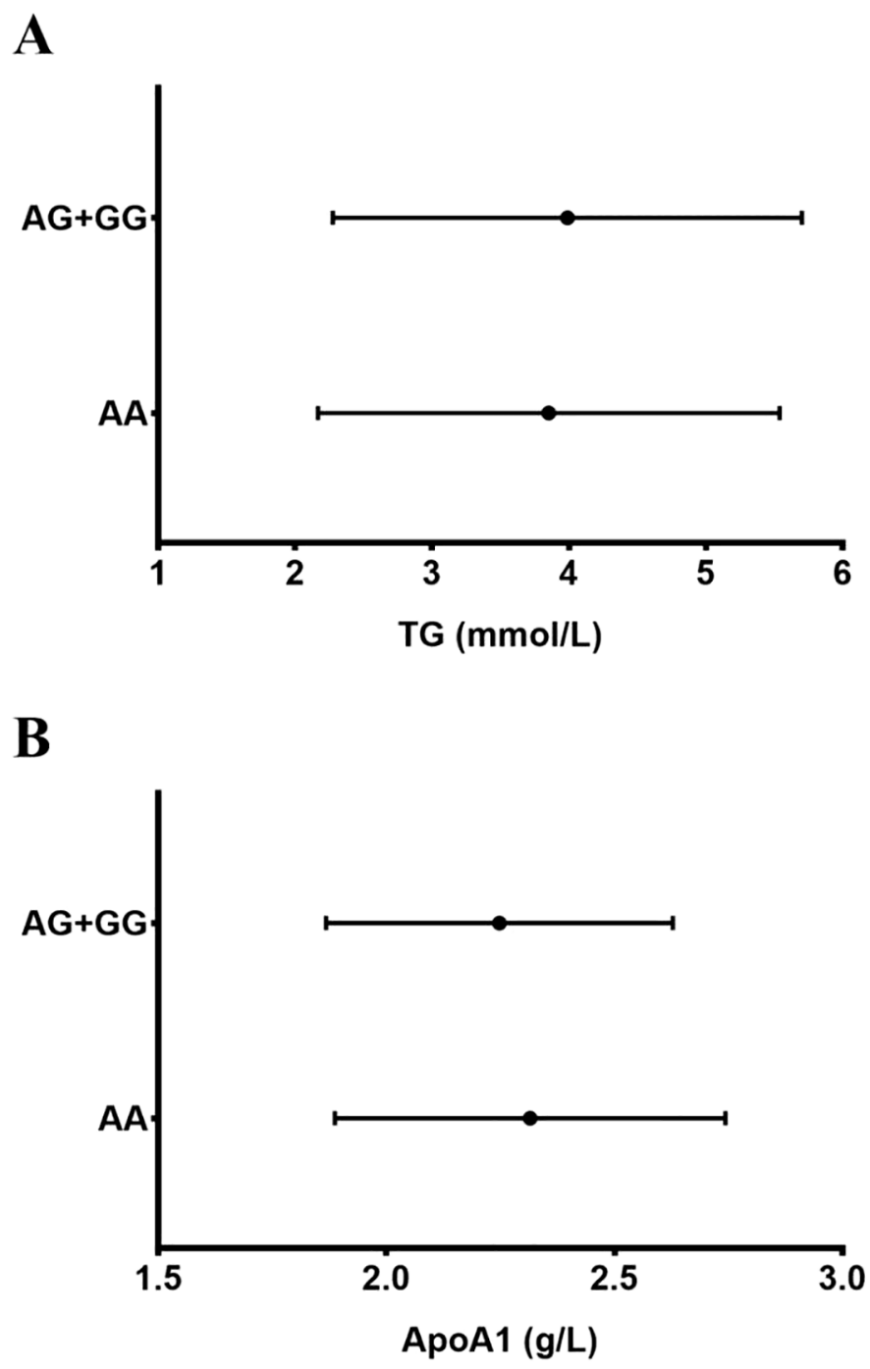
Triglyceride (TG) and ApoA1 levels according to the rs4420638 ApoC1 polymorphism (allele carrier status). A significant increase in TG **(A)** and a decrease in ApoA1 **(B)** levels in the GDM group were observed in G-allele (AG+GG) carriers compared with AA genotype carriers (P = 0.041 and 0.020, respectively). The graphs show mean values ± standard deviation (p < 0.05–0.001, by multivariable analysis).

In addition, in patients with GDM, G allele (AG+GG) carriers at the rs4420638 polymorphism had higher TG and lower apoA1 levels (both *P* < 0.05) than the AA genotype carriers (both *P* < 0.05, **Table 5**, **Figures 2A, B**). No significant variations in variables were detected when categorized according to genotype in the polymorphism among the control patients (**Table 5**).

**Table 5:** Clinical characteristics, metabolic and oxidative stress parameters of the *apoC1 rs4420638* genotypes in GDM patients and controls.

	GDM		Controls	
	AA (n=556)	AG+GG (n=168 + 10)	AA (n=820)	AG+GG (n=262 + 20)
Age (years)	35.41 ± 4.06	36.02 ± 3.95	35.40 ± 3.83	35.75 ± 3.39
Pre-pregnancy BMI (kg/m^2^)	22.25 ± 2.93	22.18 ± 2.94	21.26 ± 2.70	21.10 ± 2.66
Gestational age at sampling(week)	37.12 ± 3.18	36.31 ± 3.30 **	36.08 ± 3.52	35.93 ± 3.56
Delivery BMI(kg/m2)	26.88 ± 3.29	26.56 ± 2.76	26.77 ± 2.75	26.54 ± 2.60
Metabolic profile ^△^				
Fasting Ins (pmol/L)	107.47 ± 135.12	93.61 ± 90.55	72.09 ± 35.52	73.41 ± 35.45
Fasting Glu (mmol/L)	4.62 ± 0.75	4.62 ± 0.65	4.35 ± 0.43	4.35 ± 0.41
HOMA-IR	3.55 ± 6.12	2.87 ± 3.12	2.03 ± 1.08	2.06 ± 1.08
TG (mmol/L)	3.85 ± 1.69	4.04 ± 1.66 *	3.63 ± 1.47	3.63 ± 1.25
TC (mmol/L)	5.98 ± 1.09	5.90 ± 1.14	6.07 ± 1.07	6.09 ± 1.12
HDL-C (mmol/L)	1.98 ± 0.45	1.93 ± 0.42	2.00 ± 0.41	1.98 ± 0.41
LDL-C (mmol/L)	2.99 ± 1.00	2.94 ± 0.90	3.17 ± 0.99	3.21 ± 1.01
TG/HDL	2.07 ± 1.15	2.19 ± 1.01	1.90 ± 0.91	1.93 ± 0.78
AI	2.10 ± 0.60	2.10 ± 0.45	2.11 ± 0.59	2.15 ± 0.61
ApoA1 (g/L)	2.32 ± 0.43	2.24 ± 0.38 *	2.39 ± 0.44	2.33 ± 0.40
ApoB (g/L)	1.16 ± 0.26	1.14 ± 0.25	1.15 ± 0.26	1.16 ± 0.27
Oxidative stress parameters ^△△^				
TOS (μmol H2O2 Equiv./L)	26.15 ± 10.51	24.87 ± 10.69	21.21 ± 7.03	20.79 ± 7.01
TAC (mmol Trolox Equiv./L)	1.12 ± 0.21	1.10 ± 0.21	1.10 ± 0.19	1.11 ± 0.20
TOS/TAC	23.45 ± 10.20	22.92 ± 9.87	19.62 ± 7.35	19.23 ± 7.29
MDA (μmol/L)	5.87 ± 1.38	5.91 ± 1.52	5.39 ± 1.21	5.33 ± 1.22

Values are presented as mean ± standard deviation.

BMI: body mass index; SBP: systolic blood pressure; DBP: diastolic blood pressure; OGTT: oral glucose tolerance test; Ins: insulin; Glu: glucose; HOMA-IR: homeostatic model assessment of insulin resistance; TG: triglycerides; TC: total cholesterol; HDL-C: high-density lipoprotein cholesterol; LDL-C: low-density lipoprotein cholesterol; AI: atherogenic index; ApoA1: apolipoprotein A1; ApoB: apolipoprotein B; TOS: total oxidant status; TAC: total antioxidant capacity; MDA: malondialdehyde. All parameter comparisons were corrected for differences in age, pre-pregnancy BMI, gestational age at sampling, and fasting Ins and Glu levels between the two subgroups, except for age, pre-pregnancy BMI, and gestational age at sampling (GDM group was also corrected in insulin use).

^△^: GDM: *AA*: 525, *AG+GG*: 158 + 10; Control: *AA*: 782, *AG+GG*: 242 + 16.

^△△^: GDM: *AA*: 428, *AG+GG*: 135 + 8; Control: *AA*: 608, *AG+GG*: 193 + 14.

*: The *AG+GG* genotype compared with the *AA* genotype in the GDM group, P<0.05.

**: The *AG+GG* genotypes, compared with the *AA* genotype in the GDM group, P<0.01.

## 4 Discussion

It has been demonstrated that diabetic states lead to elevated apoC1 levels and the production of structurally modified, dysfunctional apoC1. However, establishing a direct causal relationship between total plasma apoC1 and cardiometabolic disease risk remains challenging, as this small apolipoprotein is highly exchangeable, and its biological impact depends largely on its association with either VLDL or HDL. ApoC1 has been shown to act on lipoprotein receptors by inhibiting binding mediated by apolipoprotein E, modulating the activities of several enzymes, and thus downregulates lipoprotein lipase, hepatic lipase, phospholipase A2, cholesteryl ester transfer protein, and activates lecithin-cholesterol acyltransferase. By controlling the plasma levels of lipids, apoC1 relates directly to cardiovascular physiology, and also links to other functions including inflammation and immunity, sepsis, diabetes, cancer, and viral infectivity (39). To date, few studies have examined the potential association between *apoC1* gene polymorphisms and diabetes. A small cohort study involving individuals of American Indian or Mexican descent identified the T45S structural variant of *apoC1*, which predisposes the protein to N-terminal truncation and was associated with an increased risk of obesity-related diabetes (40). Similarly, a meta-analysis revealed that the rs4420638 polymorphism was linked to an increased risk of diabetic nephropathy (41). However, a study in the Turkish population did not find a correlation between *apoC1* polymorphism (rs11568822) and diabetes (42). To the best of our knowledge, no prior studies have explored the relationship between *apoC1* polymorphisms and GDM risk, highlighting the novelty of our research.

The allele frequencies for *apoC1–*317 H1/H2 and rs4420638 genetic variants differ among ethnic groups. For *apoC1–*317 H1/H2 polymorphism, German and African American populations with H2 allele frequencies of 27.0% and 24.6%, respectively (43). This polymorphism was also shown in Itanlian, Japanese and Caribbean hispanic populations with H2 allele frequencies of 9.9%, 12.5% and 16.7%, respectively [44]. The same polymorphism was also found in the southern Han Chinese population with a H2 allele frequency of 20.0% (44). In the present study, we found that the H2 allele frequency in the Southwest Han Chinese population is 20.2%, which is lower than that reported in German and African American populations, but higher than that reported in Itanlian, Japanese and Caribbean Hispanic ethnic groups (43). In addition, the G allele frequency of the rs4420638 polymorphism in the Han Chinese was 13.7%, which is lower than that reported in UK, Swedish, Portuguese and Germany populations, with a G allele frequency of 22.1%, 19.9%, 17.3% and 16.8% (45–48), respectively, and higher than that reported in Algerian and Korean populations, with a G allele frequency of 11.0% and 11.1%, respectively (49, 50). Therefore, it is possible that the relationships between the *apoC1* genetic polymorphisms and diseases or lipoprotein levels may differ among ethnic groups.

Our results indicated that the combined H1H1/AG+GG genotype of the -317H1/H2 and rs4420638 polymorphisms was associated with an increased risk of GDM (*P* = 0.017), although neither polymorphism was independently associated with disease risk.

Several studies have demonstrated that both SNPs are associated with increased disease risk. -317 H1/H2 polymorphism (rs11568822) has been reported to be related to the risks of familial dysbetalipoproteinemia and Alzheimer’s disease (51). In addition, rs4420638 has been associated with risks of metabolic syndrome, T2DM, and coronary heart disease (28), diabetic nephropathy (52), and Alzheimer’s disease (53). In these conditions, the Ins (H2) allele of the rs11568822 and the G allele of rs4420638 (A>G variants) were frequently implicated. Our finding that H1 homozygotes combined with the G allele increased GDM risk suggests a potential interaction effect between these two SNPs. However, the underlying mechanism remains unclear and requires further investigations.

A recent study by Pirim et al. (54) in non-Hispanic White and African Black populations (NHWs and ABs) reported that *apoC1* polymorphisms, along with other variants located within the *apoE-C1-C4-C2* gene cluster region, were associated with LDL-related traits. For example, in NHWs, rs3826688 was associated with LDL-C and TC, rs1064725 with TC, and rs5112 with both LDL-C and TC. Notably, rs5112 remained statistically significant even after multivariate correction. In ABs, rs12721054 was significantly associated with LDL-C (54).

Our study found that the *apoC1* -317H1/H2 polymorphism was associated with LDL-C, TC, and apoB levels in Chinese women with GDM, aligning with these prior findings and extending them to a pregnant population. In addition, among control participants, this polymorphism was also associated with LDL-C and apoB levels but not TC, suggesting that genotype-related effects on lipid metabolism may differ in normal pregnancy versus GDM pregnant women. This highlights potential gene-environment interactions specific to the pathophysiology of GDM.

In addition to evidence that the *APOE-C1-C4-C2* gene cluster plays a central role in regulating LDL-related traits (54), recent GWAS have linked this cluster region to TG and HDL-C levels as well (55–57). Several studies have reported that the rs4420638 A/G polymorphism is associated with serum lipid profile changes, chronic inflammatory status, and increased disease risk. Specifically, the G allele has been associated with elevated TC, LDL-C, TG, and platelet-activating factor acetylhydrolase (PAF-AH) activity, as well as abdominal obesity and an increased T2DM and coronary heart disease risk (28). In the present study, G allele carriers among patients with GDM exhibited higher TG and lower apoA1 (the major protein component of HDL) levels than AA homozygotes in patients with GDM, suggesting that the G allele may contribute to adverse lipid profiles in GDM. These findings are consistent with previous reports showing *apoC1* polymorphisms to TG and HDL-C changes and provide additional evidence that the rs4420638 G allele may influence lipid metabolism in GDM. Our previous research in patients with PCOS revealed a strong linkage disequilibrium between the rs4420638 G allele and the *apoE ε4* variant (28), which may help explain its influence on lipoprotein metabolism and disease susceptibility. We speculate that a similar mechanism may apply for the G allele, with the above lipid variations, in patients with GDM. The exact reason why this gene variant affected these lipid components in our study participants remains to be further investigated. Notably, these associations were not observed in women with normal pregnancies, which may reflect inherent differences in lipid metabolism between GDM and non-GDM pregnancies.

This study also identified an association between the *apoC1–*317 H1/H2 polymorphism and atherogenic index (AI) in both GDM and control groups. The H2 allele was associated with lower AI values in the GDM and control groups (**Table 4**, *P* < 0.05 or 0.01), suggesting a potential protective role, independent of GDM status.

Several studies have demonstrated an association between oxidative stress and the pathogenesis of GDM (58). We recently reported an association between oxidative stress-related gene variations and GDM risk, such as *myeloperoxidase (MPO)* G-463A (59), *CYP2E1* C-1054T, and its combination with a 96-bp insertion/deletion (I/D) polymorphism (60). Since apoC1 is linked to oxidative stress function (58), we also evaluated whether the *apoC1* -317H1/H2 and rs4420638 polymorphisms were related to oxidative stress markers in GDM pregnancy. In the present study, we found that there was no correlation between *apoC1* genetic variants and oxidative stress markers in GDM and control participants. We speculate that the effect of the gene on these markers might not be significant, since apoC1 affecting oxidative-related traits is indirect. For example, experiments with *apoC1* rescue conditions showed decreased lipid oxidation *via* increasing PON-1 activity (61). This is in contrast with the effect of *PON-1* genetic variants on oxidative markers in GDM patients and control women in our study (62). Whether the *apoC1* genetic variants have any correlation with PON-1 activity and/or levels remains to be studied further. Additional study can clarify the potential underlying mechanism of above negative results concerning the correlation of oxidative stress markers.It is well documented that GDM is a heterogeneous disorder involving a combination of factors responsible for decreased insulin sensitivity and inadequate insulin secretion (63). The inability of pancreatic beta cells to match the increased insulin resistance to normalize the systemic glucose level translates to maternal hyperglycemia. In addition to genetic risk factors, risk factors also include advanced maternal age, family history of diabetes, previous GDM, having a macrosomic baby, race/ethnicity, being overweight or obese, cigarette smoking, diet, lifestyle, as well as environmental factors (64). Understanding these factors enhances our ability to predict, prevent, and manage GDM effectively, improving maternal and fetal health outcomes.

This study has some tentative implications for future research. The combination of the two polymorphisms predicts the association with GDM, being significant and statistically marginal. This association remains significant after multiple testing correction. This suggests that the two polymorphisms have true predisposing effect on GDM risk. However, the two individual SNPs did not predict differences in the proportion of patients with GDM. The discrepancy between the prediction of individual genotypes and clinical manifestations suggests that intragenic and polygenic factors, as well as obesity, age, environment factors, etc. common in the population, likely dominate the clinical risk modulation beyond that exerted by the single candidate gene susceptibility individual variants tested. Candidate gene studies of genetic haptotypes and multiple interacting genes, such as related genes in a common physiologic pathway (rather than single genes) may be undertaken, as suggested in a similar complex disease (CAD) (65). In addition, studies on GDM patients in combination with genotyping of -317 H1/H2 and rs4420638 SNPs may have clinical benefits in identifying risks in individuals with lipid disorders for future CAD. Large prospective trials (that include several ethnicities) are required to establish the clinical implications of our findings. This study has several strengths. First, it involved a relatively large and well-characterized Chinese cohort (n = 1836), with comprehensive metabolic and clinical data. Second, it provides linking *apoC1* to GDM and related traits, an area previously unexplored. Third, this study provides insight into the genetic regulators of lipid metabolism in the context of human pregnancy.

However, several limitations should be acknowledged. First, we did not assess apoC1 expression levels by genotypes, which limits our ability to establish functional causality. Moreover, these findings require validation in larger, multiethnic cohorts to ensure generalizability. Additionally, our analysis was limited by its observational, cross-sectional design and focused mainly on descriptive characteristics without mechanistic insights.

## 5 Conclusions

In this study, we identified for the first time that the H1H1/AG+GG genotype combination of the -317H1/H2 and rs4420638 A/G polymorphisms in the *apoC1* gene may serve as a potential genetic risk factor for GDM in Chinese women. In addition, the -317H1/H2 polymorphism was associated with TC, LDL-C, and apoB levels in patients with GDM, whereas rs4420638 A/G was linked to TG and apoA1 levels. These associations suggest a link between these variants and alterations in atherometabolic traits during GDM pregnancy. This study offers the potential to integrate the present information into genetic risk panels or risk prediction scores, which will help in the early prediction and identification of at-risk patients.

## Data Availability

The original contributions presented in the study are included in the article/**Supplementary Material**. Further inquiries can be directed to the corresponding authors.

## Supplementary material

Supplementary Figure 1 TaqMan allelic discrimination assay of the rs4420638 polymorphism in the ApoC1 gene by real-time qPCR. Different probes display different colors. HEX probe shows red, FAM probes shows blue. A: AA genotype (red). B: AG genotype (red and blue). C: GG genotype (blue).

Supplementary Figure 2 PCR-RFLP analysis of the -317H1/H2 polymorphism in the ApoC1 gene after digestion with the HpaI enzyme. The electrophoresis was performed on an agarose gel. The bands correspond to the following genetypes: H1H1 genotype: 221bp. H1H2 genotype: 221bp, 159 bp, 66 bp. H2H2 genotype:159 bp, 66 bp. Marker (M): 50bp DNA ladder.
