# Supplementary figures and images for: Apolipoprotein C1 -317H1/H2 and the rs4420638 genetic variations and risk of gestational diabetes mellitus in Chinese women: a case-control study
Source: Front Endocrinol (Lausanne). 2025 Nov 21;16:1681268. doi: 10.3389/fendo.2025.1681268 (PMC12678127; doi:10.3389/fendo.2025.1681268)

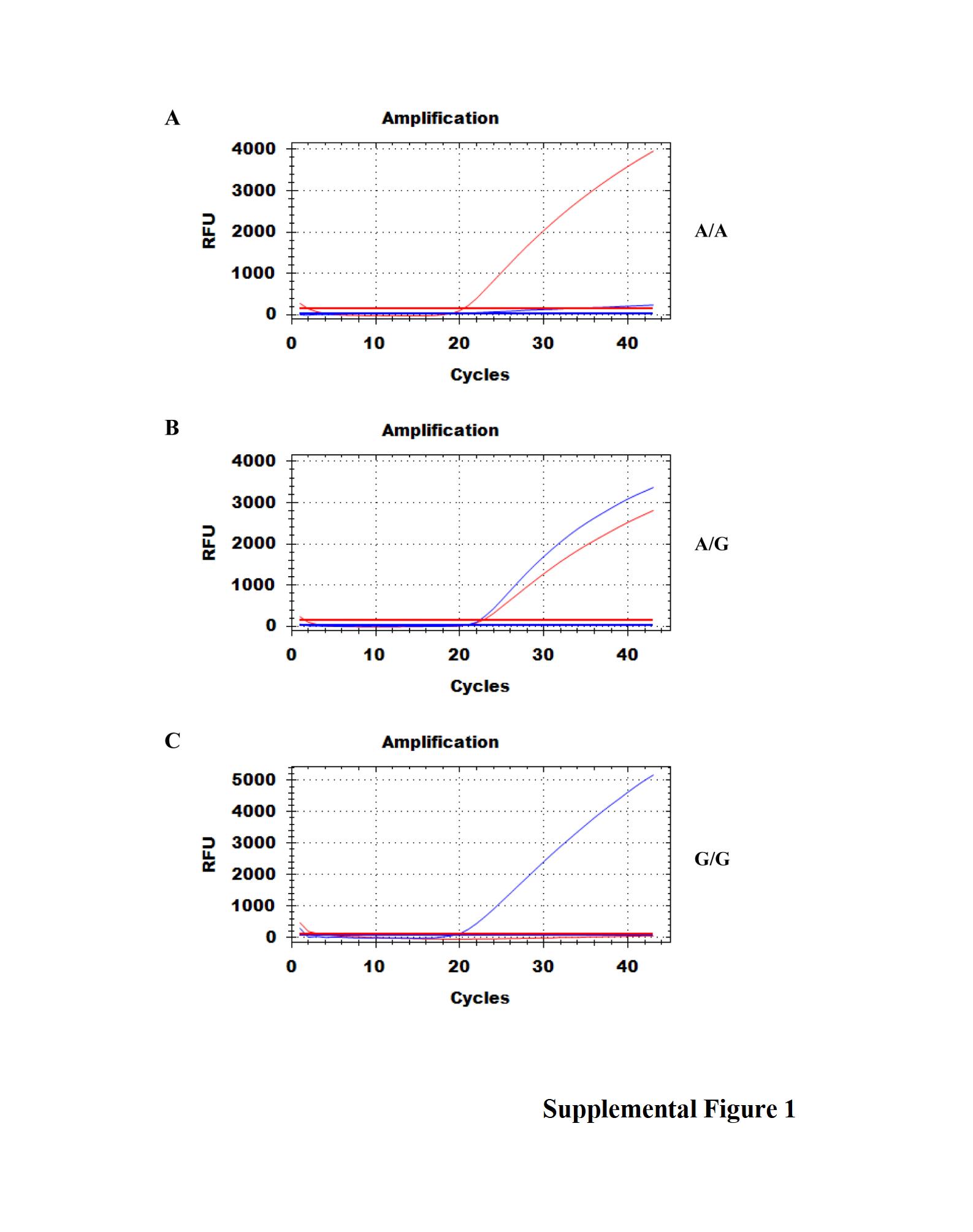

Supplement: Supplementary Figure 1 — TaqMan allelic discrimination assay of the rs4420638 polymorphism in the ApoC1 gene by real-time qPCR. Different probes display different colors. HEX probe shows red, FAM probes shows blue. A: AA genotype (red). B: AG genotype (red and blue). C: GG genotype (blue). [file Image1.jpeg]

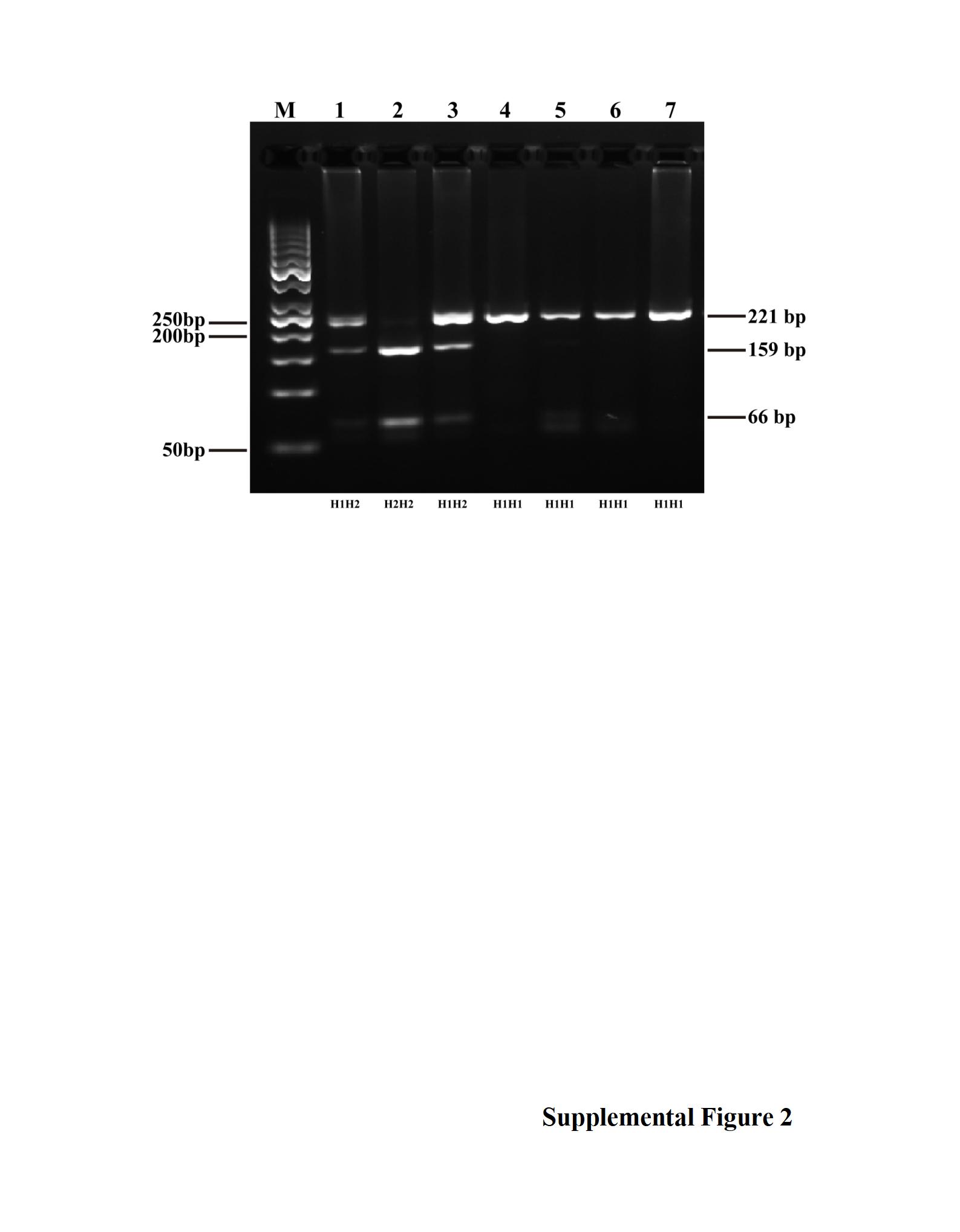

Supplement: Supplementary Figure 2 — PCR-RFLP analysis of the -317H1/H2 polymorphism in the ApoC1 gene after digestion with the HpaI enzyme. The electrophoresis was performed on an agarose gel. The bands correspond to the following genetypes: H1H1 genotype: 221bp. H1H2 genotype: 221bp, 159 bp, 66 bp. H2H2 genotype:159 bp, 66 bp. Marker (M): 50bp DNA ladder. [file Image2.jpeg]
